# Effects of Ginseng and Echinacea on Cytokine mRNA Expression in Rats

**DOI:** 10.1100/2012/942025

**Published:** 2012-05-22

**Authors:** Deniz Uluışık, Ercan Keskin

**Affiliations:** Department of Physiology, Faculty of Veterinary Medicine, University of Selcuk, 42075 Selçuklu, Konya, Turkey

## Abstract

The aim of the study was to determine the effect of ginseng and echinacea on the mRNA expression of IL-10, TNF-**α**, and TGF-**β**1 in healthy rats. Six-week-old male Fischer 344 rats (*n* = 48) were used. The animals were divided into three equal groups, as follows: control (C); ginseng (G); echinacea (E). While the C group was fed a standard rat diet (Purina) *ad libitum* for a period of 40 days, the G and E groups animals received the same diet containing 0.5 g/kg of *Panax ginseng* root powder and 0.75 g/kg of *Echinacea purpurea* root powder, respectively. Blood samples were obtained from 8 rats in each group after 20 and 40 days of treatment, and the mRNA expression of IL-10, TNF-**α**, and TGF-**β**1 was determined. After 20 days of treatment, the expression of IL-10 mRNA in the G group was different from the C group (*P* < 0.05); however, after 40 days of treatment, there was no difference between the groups. There was no difference after 20 and 40 days of treatment between the groups with respect to the expression of TGF-**β**1 mRNA. After 20 days of treatment, the expression of TNF-**α** mRNA in the E group was higher (*P* < 0.05) than the C group. After 40 days of treatment, the expression of TNF-**α** mRNA was similar in all of the groups. Based on the current study, the increase in expression of IL-10 mRNA in the G group and the increase in expression of TNF-**α** mRNA in the E group support the use of these plants for purposes of modulating the immune system. However, a more detailed study regarding the effects of ginseng and echinacea on these cytokines and other cytokines is needed.

## 1. Introduction

Ginseng refers to the root of several species in the plant genus *Panax *(C. A. Meyer Araliaceae), including *P. ginseng*, *P. japonicas*, *P. quinquefolium*, and *P. notoginseng* [1]. Recently, *P. ginseng* has been widely used worldwide as an ingredient in dietary health supplements and an additive in foods [2]. The glycosidal saponins (glycosylated steroids) known as ginsenosides are the main active components of ginseng [3]. Based on several studies, tonic, immunomodulatory, antimutagenic, and antiageing activities have been reported among the pharmacologic properties of ginseng [4, 5]. Clinical studies have also demonstrated that ginseng may improve immunostimulation, antitumor activity, cardiovascular function, antioxidant activity, hypoglycemic activity, and the pituitary-adrenocortical system [6–8].

Echinacea is another well-known herb with a worldwide reputation. There is widespread interest in the therapeutic and preventive potential of echinacea [7]. *Echinacea angustifolia*, *E. pallida*, and *E. purpurea* are the species most often used medicinally [9, 10]. It has been reported that echinacea has carbohydrate, glycoside, alkaloide, alkylamide, and polyacetylene structures as active ingredients. Echinacea is commonly used for the prevention and treatment of upper respiratory tract infections (URTIs), viral, bacterial, and fungal infections, complementary therapy for cancer chemotherapy to support the immune system, chronic fatigue syndrome, acquired immunodeficiency diseases (AIDs), and snake bites [11]. 

 In the current study, we determined the expression of IL-10 and TGF-*β*1 mRNA as antiinflammatory cytokines and TNF-*α* as a proinflammatory cytokine in healthy rats fed ginseng and echinacea. 

## 2. Materials and Methods

Ginseng and echinacea roots were commercial products (General Nutrition Products (GNC), Inc., 1050 Woodruff Road Greenville, SC, USA). In this study, 48 healthy Fischer 344 male rats were used. The rats were divided into three equal groups. The mean weights of the groups were similar. The animals were kept in individual cages for 40 days and fed *ad libitum*. The control group (C) was fed standard rat pellets, the ginseng group (G), and echinacea group (E) were fed pellets containing 0.5 g/kg of *P. ginseng* root powder and 0.75 g/kg of *E. purpurea* root powder, respectively. On the 20th and 40th days of the study, citrated blood samples were obtained from 8 animals in each group. The Ethical Committee of the Faculty of Veterinary Medicine (Report no. 2007/036) approved the study protocol. 

### 2.1. Total RNA Isolation and cDNA Synthesis

Leucocyte isolation was performed using red blood cell lysis buffer (RBCLB; Roche Diagnostics, Mannheim, Germany). Total RNA was isolated using components and instructions from the High Pure RNA Isolation Kit (Roche Diagnostics, Mannheim, Germany) in the leucocyte pellet. The total elution volume of RNA was 50 mL in the final step. RNA was stored at −80°C until used in a cDNA preparation reaction. Total RNA was used as a template for reverse transcription (RT) in 20 mL volumes, as described in the 1st Strand cDNA Synthesis Kit for RT-PCR (Roche Diagnostics, Mannheim, Germany). In the RT reaction, we used random hexamer primers. The cDNA was stored at −20°C until used in real-time PCR in a LightCycler (LC; Roche Diagnostic Ltd., Lewes, UK). 

### 2.2. Real-Time PCR

Real-time PCR was performed for the quantification of gene expression using a LC rapid thermal system (Roche) according to the manufacturer's instructions. A LightCycler FastStart DNA Master SYBR Green I kit (Roche Diagnostics, Mannheim, Germany) was used together with LightCycler-Primer Set Kits (Search-LC, Heidelberg, Germany) for IL-10, TNF-*α*, and TGF-*β*1, and a housekeeping gene (glyceraldehyde phosphate dehydrogenase (GAPDH)). Reactions were performed in a 20 *μ*L volume with 10 *μ*L of master mix (2 *μ*L of SybrGreen mix, 2 *μ*L of specific primers for each cytokine, and 6 *μ*L of sterile water) and 10 *μ*L of cDNA template. The run was programmed according to the LightCycler-Primer Set Kits instructions. The first step was a 10 min denaturation at 95°C, followed by 35 cycles for GAPDH, 45 cycles for the remaining cytokines, a 95°C denaturation for 10 sec, 68°C–58°C annealing for 10 sec (decreasing 0.5°C/cycle), and finally a 72°C extension for 16 sec. The PCR products were subjected to melting curve-analyses starting with 58°C, and increasing to 95°C (0.1°C/sec) to confirm product specificity. The results were expressed as a ratio between the concentration of the housekeeping gene (GAPDH) and the relevant cytokine in each sample. RNA input was normalized by the average expression of the housekeeping genes encoding GAPDH. 

The statistical differences among the groups were tested by REST software 2005 [12]. 

## 3. Results

**Figure 1:**
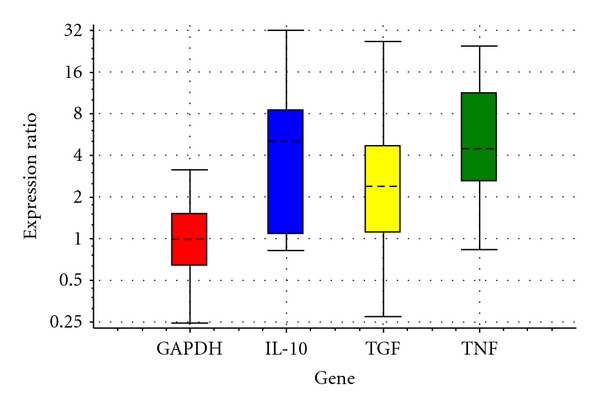
Relative expressions of IL-10, TGF-*β*1, and TNF-*α* obtained from echinacea versus control after processing RT-PCR with REST in 20th day.

**Figure 2:**
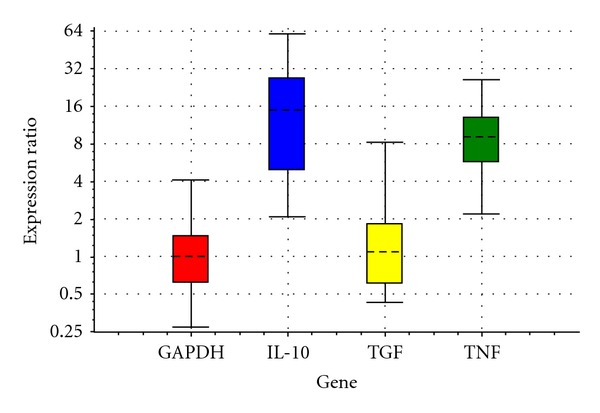
Relative expressions of IL-10, TGF-*β*1, and TNF-*α* obtained from ginseng versus control after processing RT-PCR with REST in 20th day.

**Figure 3:**
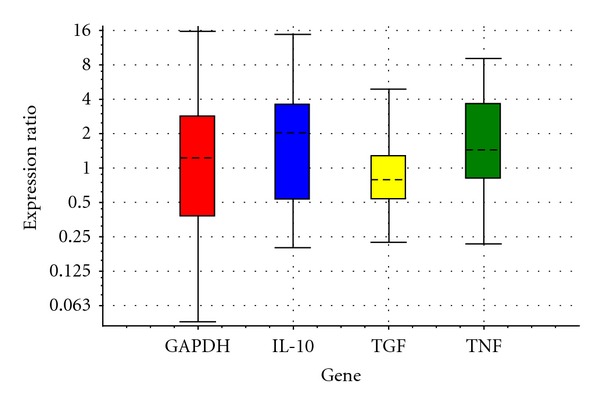
Relative expressions of IL-10, TGF-*β*1, and TNF-*α* obtained from echinacea versus control after processing RT-PCR with REST in 40th day.

**Figure 4:**
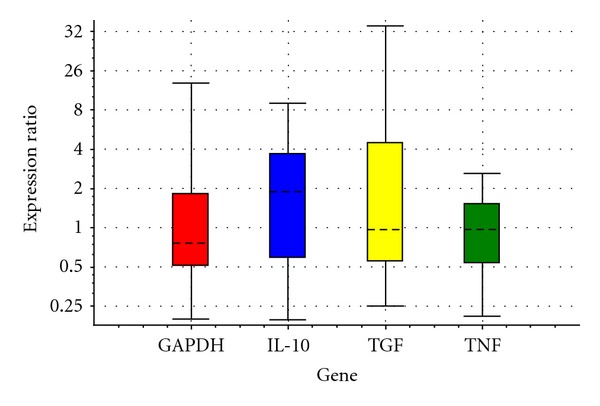
Relative expressions of IL-10, TGF-*β*1, and TNF-*α* obtained from ginseng versus control after processing RT-PCR with REST in 40th day.

In the current study, it was determined that the expression of IL-10 mRNA was not different between the C and E groups (Table 1 and Figure 1); however, the expression of IL-10 mRNA increased significantly in the G group when compared to the C group (Table 2 and Figure 2, *P* < 0.05) as a result of feeding 20 days with feeds including ginseng and echinacea. The expression of IL-10 mRNA on day 40 of treatment was not different between the groups (Tables 3 and 4 and Figures 3 and 4). It was also shown that the expression of IL-10 mRNA in each group on days 20 and 40 of treatment was not statistically different. 

There was no difference between the groups with respect to the expression of TGF-*β*1 mRNA on the 20th and 40th days of treatment (Tables 1, 2, 3, and 4 and Figures 1, 2, 3, and 4). It was also shown that the expression of TGF-*β*1 mRNA in each group on days 20 and 40 of treatment was not statistically different. 

The expression of TNF-*α* mRNA in the E group on the 20th day of treatment was significantly higher than the C group (Table 1 and Figure 1, *P* < 0.05). In addition, there was no significant difference between groups with respect to the expression of TNF-*α* mRNA on the 40th day of treatment (Tables 3 and 4 and Figures 3 and 4). In contrast, the expression of TNF-*α* mRNA obtained on days 20 and 40 of treatment did not differ significantly for the 3 groups as a function of time. 

## 4. Discussion

The administration of ginseng for 20 days increased the expression of IL-10 mRNA significantly (*P* < 0.05) when compared to the C group (Table 2 and Figure 2), and this increase was in agreement with the findings obtained by Liou et al. [13] and Wang et al. [14] using mouse cell cultures in which a ginseng extract was used. 

In contrast, Larsen et al. [15] reported that ginseng extract did not change IL-10 production in polymorphonuclear leukocytes, while Lee et al. [16] showed that the addition of ginseng extract in human monocytic cell cultures decreased the expression of IL-10. The equilibrium that should exist between pro- and anti-inflammatory cytokines in various infections and inflammatory states is critical with respect to host defense. Indeed, an uncontrolled and excessive immune response may result in more harm than an infection or various noxious agents cause in humans [17]. The increase in the expression of IL-10 and suppression of IL-2 production in T cells following ginseng application and the corresponding limited proliferation in B and T cell lines and decrease in immunoglobulin production in B cells support this opinion [13]. 

In addition, it is difficult to explain the lack of difference in IL-10 mRNA expression in the G group compared to the C group on day 40 of treatment (Table 4 and Figure 4), because the effect of ginseng treatment on the expression of IL-10 mRNA as a function of time, if any, is unknown. 

The expression of IL-10 mRNA of the E group on the 20th day of the study was not different from the C group (Table 1 and Figure 1). While Gertsch et al. [18] defined that the addition of echinacea extract in human peripheral mononuclear cell cultures increased IL-10 expression, Hwang et al. [19] reported that the addition of echinacea to mouse splenocyte cell cultures increased IL-10 expression, and Raduner et al. [20] concluded that the addition of echinacea alkylamide to human peripheral whole blood cell cultures increased the expression of IL-10. In contrast, Guiotto et al. [21] showed that oral echinacea in various doses in humans decreased the expression of IL-10 and Zhai et al. [22] reported that there was no difference in the expression of IL-10 by LPS stimulation in mononuclear leucocyte cell cultures obtained from mice that had received an oral echinacea extract when compared to the control group. Echinacea or alkylamides first stimulate a proinflammatory response, then an antiinflammatory response; this time-dependent bimodal effect is important in immunomodulation [23]. 

While variations in TGF-*β* mRNA following echinacea treatment were not observed in the current study, Randolph et al. [23] reported that the expression of TGF-*β* mRNA decreased in cell cultures which were treated with different echinacea extracts (*E. purpurea *and *E. angustifolia*). While a significant increase or decrease was not observed in the TGF-*β* level following ginseng treatment in the current study, different statements suggested that the TGF-*β*1 level increased or decreased in the research conducted with single or combined application of various saponins that are active and contained in this plant or in the root of the plant. 

Kanzaki et al. [24] stated that ginseng saponin application in human fibroblast cultures increased the TGF-*β*1 level. An increase in TGF-*β*1 depends on saponin; therefore, the materials effective in extracellular matrix formation, such as fibronectin, may be beneficial in tissue repair and wound healing [24]. In contrast, Han et al. [25] concluded that ginseng administered to mice intraperitoneally 24 hours before irradiation decreased the expression of TGF-*β*1 mRNA and affected antioxidant activity positively; Han et al. [25] supported their opinion based on the findings of Chang et al. [26] and Martin et al. [27] in which TGF-*β* increased radicals, such as ROS and HO, causing oxidative damage. 

While ginseng did not cause a significant alteration in the expression of TNF-*α* mRNA in this study, Wang et al. [28] stated that a ginseng extract did not result in any alterations in the expression of TNF-*α* mRNA in mouse macrophages. In contrast, Rivera et al. [29] reported that ginsenoside Rb1 increased TNF-*α* titration in lymphocyte cultures obtained from mice after parvovirus inoculation, while Song et al. [30] concluded that there was an increase in the expression of TNF-*α* mRNA by ginseng extract application in murine peritoneal macrophage cell cultures. In contrast, Huang et al. [31] stated that the addition of ginseng extract in human lymphocyte cell cultures decreased the expression of TNF-*α*, and Kim et al. [32] showed that the addition of ginsenoside Rb1 to human peripheral mononuclear cell cultures decreased the expression of TNF-*α* and there was also a decrease in the expression of TNF-*α* when given orally to mice with arthritis. In these studies with ginseng, different results and decisions should be evaluated with various ginseng content, different systems, and varying amounts. 

Randolph et al. [23] and Gertsch et al. [18] stated that the addition of echinacea extract to human monocytic cell cultures and echinacea alkylamide to human macrophage cell cultures increased the expression of TNF-*α* mRNA, which supported the increase in the expression of TNF-*α* mRNA in the E group in the current study. In parallel with these findings, it was determined that echinacea root powder or extracts increased the TNF-*α* level in cell cultures treated with LPS [22] and in infected cell cultures [33]. In contrast, oral echinacea in humans in various doses [21] and the addition of echinacea extract to human peripheral whole blood cultures [20] decreased the TNF-*α* level. 

In the studies which showed that echinacea extracts or preparations decreased or increased cytokines, such as TNF-*α*, it was suggested that this material modulated the immune system both as a stimulator and as an inhibitor via CB2 receptors [18]. It was determined that the different results obtained with the echinacea studies were correlated with the content from different kinds and parts of this plant [20]. 

When the increase in the expression of IL-10 mRNA by ginseng was taken into consideration in this study, it can be concluded that this plant is effective in immunomodulation. In the E group of this study, the increase in the expression of TNF-*α* mRNA was considered with some cytokines (IL-1, IL-6, NO, and IFN-*γ*) not measured in our study, supporting the opinion that echinacea modulates and stimulates the immune system. Because no variations occurred in the expression of TGF-*β*1 mRNA by ginseng or echinacea in the amounts used in the current study, it cannot be concluded that these plants have no effect on TGF-*β*1. 

Given the complexity of the immune system, the interaction with other systems and the multifunctionality of measured cytokines were taken into consideration, more detailed and different studies should be conducted to confirm the results. 

## Figures and Tables

**Table 1:** Statistical analysis summary of results obtained from echinacea versus control after processing RT-PCR data through REST (Relative Expression Software Tool) in 20th day.

Gene	Type	Reaction efficiency	Expression	Std. error	95% CI	P (H1)	Result
GAPDH	REF	0,91	1,000	0,501–2,050	0,313–2,909	1,000	
IL-10	TRG	0,91	4,053	0,956–12,147	0,867–22,956	0,131	
TGF-*β*1	TRG	1,0	2,569	0,715–8,246	0,334–19,656	0,472	
TNF-*α*	TRG	0,91	5,042	2,078–13,565	0,944–23,456	0,035	UP

Normalisation factor of the parameters used for analysis was 0.40.

**Table 2:** Statistical analysis summary of results obtained from ginseng versus control after processing RT-PCR data through REST (Relative Expression Software Tool) in 20th day.

Gene	Type	Reaction efficiency	Expression	Std. error	95% CI	P (H1)	Result
GAPDH	REF	0,91	1,000	0,497–1,970	0,295–3,419	1,000	
IL-10	TRG	0,91	12,304	4,545–33,116	2,140–61,238	0,041	UP
TGF-*β*1	TRG	1,0	1,179	0,556–2,755	0,464–4,988	1,000	
TNF-*α*	TRG	0,91	8,468	4,847–19,217	2,357–24,843	0,065	

Normalisation factor of the parameters used for analysis was 0.18.

**Table 3:** Statistical analysis summary of results obtained from echinacea versus control after processing RT-PCR data through REST (Relative Expression Software Tool) in 40th day.

Gene	Type	Reaction efficiency	Expression	Std. Error	95% CI	P (H1)	Result
GAPDH	REF	0,91	1,000	0,270–3,369	0,086–11,659	1,000	
IL-10	TRG	0,91	1,707	0,369–5,595	0,233–11,811	0,263	
TGF-*β*1	TRG	0,91	0,837	0,404–1,686	0,248–3,303	0,508	
TNF-*α*	TRG	0,91	1,587	0,583–4,967	0,307–8,978	0,241	

Normalisation factor of the parameters used for analysis was 1.00.

**Table 4:** Statistical analysis summary of results obtained from ginseng versus control after processing RT-PCR data through REST (Relative Expression Software Tool) in 40th day.

Gene	Type	Reaction efficiency	Expression	Std. Error	95% CI	P (H1)	Result
GAPDH	REF	0,91	1,000	0,443–2,742	0,230–7,522	1,000	
IL-10	TRG	0,91	1,533	0,394–4,632	0,200–6,599	0,502	
TGF-*β*1	TRG	0,91	1,522	0,446–8,461	0,263–25,741	0,565	
TNF-*α*	TRG	0,91	0,897	0,463–1,689	0,246–2,596	0,876	

Normalisation factor of the parameters used for analysis was 1.46.
